# Mechanochemical ablation (MOCA) for superficial venous insufficiency: a protocol for a systematic review with meta-analysis

**DOI:** 10.1590/1677-5449.202301432

**Published:** 2026-02-18

**Authors:** Simone Pedroso Jardim, Vinicius Farina Sartori, Iana Kátia Araújo Gonçalves, Carolina Dutra Queiroz Flumignan, Jorge Eduardo de Amorim, Ronald Luiz Gomes Flumignan, Luis Carlos Uta Nakano

**Affiliations:** 1 Universidade Federal de São Paulo – Unifesp, São Paulo, SP, Brasil.; 2 Centro Universitário São Camilo, São Paulo, SP, Brasil.

**Keywords:** varicose veins, veins, saphenous vein, ablation techniques, sclerosis, sclerosing solutions, sclerotherapy, review, evidence-based medicine, meta-analysis, varizes, veias, veia safena, técnicas de ablação, esclerose, soluções esclerosantes, escleroterapia, revisão, medicina baseada em evidências, metanálise

## Abstract

Varicose veins are a common disease worldwide, mainly affecting adults, and are estimated to be the 7th most common reason for physician referral in the USA. There is no ideal technique for treatment of varicose veins. Several techniques have emerged in recent years: the most widely used are thermal techniques such as laser and radio frequency and non-thermal techniques such as chemical and mechanochemical ablation (MOCA). MOCA employs a combination of physical damage to the vessel with infusion of a sclerosant fluid with the objective of improving the effects and avoiding disadvantages of thermal ablation such as pain and nerve injuries. The aim of this study will be to evaluate the effects of MOCA for treatment of superficial varicose veins in the lower limbs. We will search randomized controlled trials of MOCA for treatment of varicose veins. The search strategy will include free text terms and controlled vocabulary terms (e.g. Emtree, MeSH) for ‘varicose veins’, ‘varices’, ‘ablation’, ‘mechanical ablation’, ‘chemical ablation’, and ‘mechanochemical ablation’. We will search at least the following databases: Medline (via Pubmed), Embase, Cochrane Central Register of Controlled Trials, Lilacs, Ibecs, WHO Clinical Trials Platform, and Clinicaltrials.com. The risks of bias will be evaluated with the Cochrane tool. We will report structured summaries of the included studies and conduct meta-analyses if possible. Development of new treatments such as MOCA must be encouraged and validation by systematic reviews is required to demonstrate their effects and support better clinical treatment decisions.

## Resumo

As varizes são uma das doenças mais comuns no mundo, afetando principalmente adultos, e estima-se que representem o sétimo motivo mais frequente de encaminhamento médico nos EUA. Embora não haja consenso sobre a técnica ideal para o tratamento de varizes, várias abordagens surgiram nos últimos anos. As técnicas térmicas, como *laser* e radiofrequência, e as técnicas não térmicas, como ablação química e mecanoquímica (MOCA), estão entre as mais usadas. A MOCA combina o dano físico ao vaso com a infusão de uma solução esclerosante, buscando potencializar os efeitos e evitar as desvantagens associadas às técnicas térmicas, como dor e lesões nervosas. O objetivo deste estudo é avaliar os efeitos da MOCA no tratamento de veias varicosas superficiais nos membros inferiores. Serão incluídos ensaios clínicos randomizados e controlados que investiguem a técnica para o tratamento de varizes. A estratégia de pesquisa abrangerá termos controlados (por exemplo, Emtree, MeSH) e de texto livre relacionados a “varizes”, “ablação”, “ablação mecânica”, “ablação química” e “ablação mecanoquímica”. Serão consultadas, no mínimo, as seguintes bases de dados: Medline (via PubMed), Embase, Cochrane Central Register of Controlled Trials, LILACS, IBECS, WHO Clinical Trials Platform e Clinicaltrials.com. Os riscos de viés serão avaliados por meio da ferramenta da Cochrane. Serão apresentados resumos estruturados dos estudos incluídos e, quando possível, realizadas metanálises. O desenvolvimento de novos tratamentos, como a MOCA, deve ser incentivado, e sua validação por meio de revisões sistemáticas é essencial para demonstrar seus efeitos e orientar uma melhor tomada de decisão clínica.

## Introduction

Varicose veins are a common disease worldwide. European studies have shown high prevalence of varicose veins in the general population.^1,2^ In the United States, varicose veins are also a very common disease in adult populations and are estimated to be the 7th most common reason for physician referral.^3^ The annual incidence of varicose veins estimated in the Framingham Study was 2.6% among women and 1.9% among men and did not vary within the age range studied (40-89 years).^4^ Varicose veins can lead to chronic venous disease, which has serious consequences such as absence from work, pain, cosmetic and psychological damage, and reduced quality of life.^5^ The socioeconomic impact of chronic venous insufficiency is an underestimated public health problem.^6^

There is no ideal technique for treatment of varicose veins. Several techniques have emerged in recent years: the most widely used are thermal techniques such as laser and radio frequency and non-thermal techniques such as chemical and mechanochemical ablation (MOCA).^7^

MOCA employs a combination of physical damage to the vessel and infusion of a sclerosant fluid, with the objective of improving the effects and avoiding disadvantages of thermal ablation, such as pain and nerve injuries. The MOCA technique is performed using a catheter placed within the vein. The catheter deploys a rotating hollow wire that causes physical damage to the endothelium, causing the veins to go into spasm. At the same time, the physician injects a chemical agent through the hollow wire into the vein, which results in protein denaturation, endothelial destruction, and endoluminal fibrosis.^8^ Several studies have reported technical efficacy from 87 to 97%.^9-11^ In contrast to thermal ablation, the MOCA technique does not involve heat or tumescent anesthesia. Consequently, MOCA can reduce heat-related complications such as pain, hematoma, induration, and nerve injury.^8^

Previous studies suggested that MOCA is associated with lower procedure times, lower postprocedural pain, and faster recovery than endovenous thermal ablation techniques.^12^ This review is important to show the effectiveness of MOCA for the treatment of varicose veins and compare it with existing techniques. The aim of this systematic review will be to evaluate the effects of mechanochemical ablation (MOCA) for the treatment of superficial varicose veins in the lower limbs. Despite the limited number of studies conducted in Brazil, highlighting the MOCA® technique is highly relevant to the current Brazilian context, where access to less invasive procedures with fewer postoperative complications is increasingly valued, especially in public and supplementary health care systems seeking to reduce recovery times and improve cost-effectiveness.

Moreover, the latest guidelines from the Brazilian Society of Angiology and Vascular Surgery (SBACV), published in 2023, include MOCA® as one of the techniques recommended for treatment of great saphenous vein insufficiency. The guideline highlights MOCA as a viable alternative, particularly for patients who wish to avoid thermal methods or tumescent anesthesia, and emphasizes its benefits in terms of reduced postoperative discomfort and quicker return to daily activities. This reinforces the clinical relevance of MOCA in the Brazilian setting and justifies the need for further studies in Brazil and for discussions of its implementation and long-term outcomes.

Moderate- to low-quality evidence exists showing that there is less recanalization or persistence of reflux and recurrence of reflux at 1 year after endovascular laser compared with conventional surgery for treatment of small saphenous veins. There is not yet certainty with relation to which is the superior technique for treatment of small saphenous veins between ultrasound guided foam sclerotherapy or conventional surgery.^13^ There are also no systematic reviews addressing MOCA for all superficial varicose veins or for great saphenous vein insufficiency specifically.

## Methods AND ANALYSIS

This protocol has been registered on the ‘International prospective register of systematic reviews’ (PROSPERO) under number CRD42017055127 and has been approved by the Ethics Committee at the Universidade Federal de Sao Paulo, Brazil (CAAE: 640.79417.5.0000.5505). We have written the protocol in accordance with the Cochrane Handbook for Systematic Reviews of Interventions^14^ and will report it in accordance with the ‘Preferred Reporting Items for Systematic Review and Meta-Analysis Protocols’ (PRISMA-P).^15^

### Types of studies

For this systematic review, we will only include randomized controlled trials (RCTs). Other designs will not be considered for the purpose of this review.

### Types of participants

Patients will be considered with varicose veins of lower limbs, with no limits set for age, gender, or sex. A diagnosis of varicose veins will be accepted if performed by clinical examination (physician) and objectively confirmed with at least one additional examination: duplex ultrasound (at least 0.5 s of reflux time in superficial veins measured in an upright position) or angiography (by computed tomography, magnetic resonance imaging, or digital subtraction). Participants will be the unit of analysis and will be analyzed using an intention-to-treat approach. Whenever available, data regarding the baseline diameter (caliber) of the treated varicose veins will also be extracted and analyzed, since vein caliber may represent an important confounding factor when evaluating primary outcomes such as occlusion rate. This will enable better stratification and interpretation of the results.

### Types of interventions

The interventions considered will be any type of MOCA (e.g. any type of mechanical device and any type of chemical agent) for the intervention groups, either in isolation or compared to placebo, to no intervention, or to a different type of ablation (e.g. endovenous laser or radiofrequency ablation), or chemical ablation alone (e.g. sclerotherapy), or conventional surgery. We will also consider any MOCA as adjunctive therapy if we find RCTs of main therapy with MOCA vs. main therapy with placebo, no intervention, or a different type of MOCA. In each situation (in isolation or adjunctive) we will consider studies with any dose of chemical sclerosing agent and any duration of intervention.

### Types of outcome measures

#### Primary outcomes

Our primary outcomes will include: (1) pain after procedure, by normalized scale or other validated methods; (2) primary occlusion rate, the proportion of participants with successful varicose vein occlusion up to 6 weeks after intervention; (3) safety outcomes; the proportion of patients with at least one serious adverse event (i.e., those that are immediately life-threatening, or resulted in hospitalization, incapacity, malignant disease, nerve injury, pulmonary embolism, or death).

Pain will be considered if it is evaluated by any validated method: e.g. Visual Analogue Scale or numbered pain score on a scale of 0 to 10.^16,17^

Primary occlusion rate will be considered according to data on occlusion of varicose veins treated by the intervention if occlusion was confirmed by an objective method (e.g. duplex ultrasound or any type of angiography). We will consider superficial vein reflux to be pathological if it exceeds 0.5 s on duplex ultrasound.

All information about adverse events will be reported and the serious adverse events listed above will be treated as a primary outcome.

We plan to assess these outcomes at 6 weeks, 1 year, and annually thereafter, grouping trials that fall within these time points (e.g., grouping trials that assess the outcomes up to 6 weeks).

#### Secondary outcomes

We will assess the proportion of patients with: (1) recurrent vein reflux, i.e. participants with significant superficial vein reflux 6 weeks after the intervention and (2) quality of life. We will also evaluate quality of life measured by any validated questionnaire or by related information such as days before return to work; and (3) costs will also be considered as an outcome if data are available.

These outcomes will be assessed at the same time points as the primary outcomes.

### Methods for literature search

#### Electronic searches

We will search the following databases: Embase (via Elsevier), Medical Literature Analysis and Retrieval System (Medline via Pubmed), Cochrane Central Register of Controlled Trials (via Wiley), Latin American and Caribbean Center on Health Sciences Information (Lilacs) and Índice Bibliográfico Español de Ciencias de la Salud (IBECS). The search strategy will include controlled terms, e.g., MesH, Emtree and also free-text terms related to ‘varicose veins’, ‘varices’, ‘ablation’, ‘mechanical ablation’, ‘chemical ablation’, and ‘mechanochemical ablation’. No limits will be set for language, date, or status of the publication. We will also search the clinical trial registries Clinicaltrials.gov and the World Health Organization International Clinical Trials Registry Platform and the gray literature source OpenGrey.

#### Manual search

We will examine the reference lists of all included studies and review articles for additional RCTs. We will contact the authors of identified trials and ask them about additional data from included RCTs and for other published and unpublished studies. We will also contact manufacturers and specialists in the field of vascular surgery for additional studies.

### Selection of studies

Two review authors (LCUN and RLGF) will independently evaluate trials to determine if they are appropriate for inclusion. We will resolve disagreements by discussion within the review authors team.

We will read the full-text articles for potentially eligible studies before finally deciding on those to include and giving reasons for all exclusions at this stage. We will record the selection process in sufficient detail to complete a PRISMA flow diagram^15^ and a characteristics of excluded studies table.^14^

### Data extraction and management

Two review authors (LCUN and RLGF) will independently extract the data from included RCTs. We will use a standard data collection form to extract study characteristics and outcome data. All aspects related to the following five main parameters will be evaluated, as per the Cochrane handbook.^14^

- Methods: study design, the total duration of study and period when carried out, number and location of study centers, study setting, dropouts, and date of study;
- Participants: number, age aspects (i.e. mean, range, etc.), gender, the severity of the condition, diagnoses, and inclusion and exclusion criteria;
- Interventions: intervention, comparison, concomitant medications, and excluded medications;
- Outcomes: primary and secondary outcomes (the final outcomes reported and those planned), and time points reported;
- Notes: funding for trial and notable conflicts of interest of trial authors.

One of the authors will plot data on Review Manager software for statistical analysis.^18^

### Assessment of risk of bias in included studies

Two review authors (LCUN and RLGF) will independently assess the risk of bias using Cochrane’s ‘Risk of Bias’ tools, as described in Section 8.5 of the Cochrane Handbook for Systematic Reviews of interventions.^14^ We will resolve disagreements by discussion within the review team. The tool covers seven domains: (1) random sequence generation, (2) allocation concealment, (3) blinding of participants and personnel, (4) blinding of outcome assessment, (5) incomplete outcome data, (6) selective outcome reporting, and (7) other bias. Each domain will be judged as high risk, low risk, or unclear risk of bias according to the criteria described in the risk of bias table in the Cochrane Handbook.^14^ We will consider blinding separately for different key outcomes when necessary. When considering treatment effects, we will take into account the risk of bias of the studies that contributed to that outcome.

### Measures of treatment effect

We will use risk ratio for dichotomous data and mean difference for continuous data with identical scales or standardized mean difference for continuous data with different scales. For all data, we will consider 95% confidence intervals.

### Unit of analysis issues

We will consider each lower limb treated as the unit of analysis for all outcomes for which it is appropriate (i.e. primary occlusion rate and recurrent vein reflux). However, we will consider the participant as the unit of analysis for other outcomes such as pain, adverse events, quality of life, and costs. We will use the intention-to-treat approach.

### Dealing with missing data

We will contact the authors or study sponsors of all included studies to verify details of data on characteristics or to obtain missing numerical outcome data when possible. We will not consider that important bias is present if outcome data are missing for both intervention groups, but reasons for these omissions are both reported and balanced across groups. However, we will consider that important bias is present if the reasons have different implications for the groups compared. In dichotomous studies, the potential impact of missing data depends on the frequency or risk of outcomes. For continuous outcomes, the potential impact increases with the proportion of participants with missing data.^14^

### Assessment of heterogeneity

We will assess studies for clinical and methodological heterogeneity. We will assess heterogeneity using the I^2^ statistic (I^2^ = ((Q - df)/Q) x 100% where Q is the chi-square statistic, and ‘df’ is degrees of freedom). If studies are deemed homogeneous according to these criteria, we will conduct meta-analyses and analyze statistical heterogeneity by visual inspection of forest plots. We will use these ranges to guide our interpretation of the I^2^ statistic: 0% to 25% will indicate low heterogeneity, 25% to 75% will indicate moderate heterogeneity, and more than 75% will indicate substantial heterogeneity.^19^ This illustrates the percentage of variability in effect estimates resulting from heterogeneity rather than sampling error.^14^ In cases of substantial heterogeneity, we will try to explain heterogeneity in terms of the pre-specified groups, for the subgroup analysis and also in terms of the possible findings of the assessment of publication bias. Additionally, we will explore whether the baseline diameter (caliber) of the treated varicose veins influences the primary outcome measures, such as occlusion rate. When this information is reported, subgroup analyses or meta-regression may be used to assess the impact of vein caliber as a potential confounding variable.

### Assessment of reporting biases

We will assess the presence of publication bias and other reporting biases using funnel plots if sufficient studies (more than 10) are included in the meta-analysis.^14,19,20^

### Data synthesis

We will synthesize data using Review Manager software and perform meta-analyses whenever possible.^18^ We will use the fixed-effects model to synthesize the data if there are low to moderate levels of heterogeneity. If there is substantial heterogeneity we will use a random-effects model. If there is considerable heterogeneity we will not undertake a meta-analysis but will describe the data in the text.

### Subgroup analyses and investigation of heterogeneity

If there are sufficient data available for the primary outcomes, we will perform subgroup analyses for the following variables: participant characteristics (age, gender, and race) and intervention characteristics (types of sclerosants, laser, and combinations of methods). One important consideration is the potential influence on outcomes such as occlusion rate of the baseline caliber of the treated veins. Larger diameter veins may respond differently to mechanochemical ablation, possibly affecting treatment success and recurrence rates. Therefore, interpreting the results while accounting for this anatomical variability is essential to enhance the clinical applicability of our conclusions.

### Sensitivity analysis

Sensitivity analyses will be conducted to determine the impact of exclusion of studies with overall high risk of bias. We plan to exclude, from a separate meta-analysis of treatments, studies with a high risk of bias in at least one of the main domains of the Risk of Bias tool (generation of randomization sequence, allocation concealment, or blinding).^21^

### Summary of findings table

We will use the ‘Grading quality of evidence and strength of recommendations for diagnostic tests and strategies’ (GRADE) approach to interpreting the findings of the review.^21^ We will construct a ‘Summary of findings’ table for all primary outcomes ([1] pain after the procedure; [2] primary occlusion rate and [3] adverse events), using GRADEpro software.^22^ We will assess the quality of the body of evidence by considering the overall risk of bias of the included studies, the directness of the evidence, the inconsistency of the results, the precision of the estimates, and the risk of publication bias.^21^ In accordance with GRADE recommendations,^23^ we will present ‘Summary of findings’ results for a single time point. We will base this table on methods described in Chapter 11 and Chapter 12 of the Cochrane Handbook for Systematic Reviews of Interventions.^14^ We will justify any departures from the standard methods.

## Results

The results are not available because this is a registered protocol for a systematic review. We are currently in the process of executing the search strategy and selecting the studies for the review. All methodological differences between the protocol and the final review will be reported.

### Ethics and dissemination

There are a great number of techniques for the treatment of varicose veins. MOCA is a recent technique the effects of which are not yet well established. There are no systematic reviews that address clinical decision-making for all superficial veins or for great saphenous vein insufficiency specifically. Therefore, we intend to perform a high-quality systematic review to show the best evidence on the effects of MOCA for treatment of varicose veins. The Ethics Committee at the Universidade Federal de Sao Paulo, Brazil approved this protocol under CAAE number 640.79417.5.0000.5505, Substantiated Opinion number 1.923.007, and PROSPERO Registration number CRD42017055127. We plan to publish all the results of this protocol for a systematic review.

## Data Availability

Data not reported or used: “Data sharing does not apply to this article, as no data were generated or analyzed”.
